# A prospective observational study of associated anomalies in Hirschsprung’s disease

**DOI:** 10.1186/1750-1172-8-184

**Published:** 2013-11-23

**Authors:** Alessio Pini Prato, Valentina Rossi, Manuela Mosconi, Catarina Holm, Francesca Lantieri, Paola Griseri, Isabella Ceccherini, Domenico Mavilio, Vincenzo Jasonni, Giulia Tuo, Maria Derchi, Maurizio Marasini, Gianmichele Magnano, Claudio Granata, Gianmarco Ghiggeri, Enrico Priolo, Lorenza Sposetti, Adelina Porcu, Piero Buffa, Girolamo Mattioli

**Affiliations:** 1 Department of Pediatric Surgery, Istituto Giannina Gaslini, Largo G. Gaslini, 5, 16100 Genoa, Italy; 2 DINOGMI, Università di Genova, Genova, Italy; 3 Department of Health Science, Biostatistics Section, Università di Genova, Genova, Italy; 4 UOC Medical Genetics, Istituto Giannina Gaslini, Genoa, Italy; 5 Unit of Clinical and Experimental Immunology, Humanitas Clinical and Research Center, Rozzano, Milan, Italy; 6 Department of Medical Biotechnologies and Translational Medicine, University of Milan, Milan, Italy; 7 Cardiovascular Department, Istituto Giannina Gaslini, Genoa, Italy; 8 Radiology Unit, Istituto Giannina Gaslini, Genoa, Italy; 9 Nephrology Unit, Istituto Giannina Gaslini, Genoa, Italy; 10 Ophthalmology Unit, Istituto Giannina Gaslini, Genoa, Italy; 11 Otorhinolaryngology Unit, Giannina Gaslini Institute, Genoa, Italy

**Keywords:** Hirschsprung, Phenotype, Observational study, Associated anomalies

## Abstract

**Background:**

Associated anomalies have been reported in around 20% of Hirschsprung patients but many Authors suggested a measure of underestimation. We therefore implemented a prospective observational study on 106 consecutive HSCR patients aimed at defining the percentage of associated anomalies and implementing a personalized and up-to-date diagnostic algorithm.

**Methods:**

After Institutional Ethical Committee approval, 106 consecutive Hirschsprung patients admitted to our Institution between January 2010 and December 2012 were included. All families were asked to sign a specific Informed Consent form and in case of acceptance each patient underwent an advanced diagnostic algorithm, including renal ultrasound scan (US), cardiologic assessment with cardiac US, cerebral US, audiometry, ENT and ophthalmologic assessments plus further specialist evaluations based on specific clinical features.

**Results:**

Male to female ratio of our series of patients was 3,4:1. Aganglionosis was confined to the rectosigmoid colon (classic forms) in 74,5% of cases. We detected 112 associated anomalies in 61 (57,5%) patients. The percentage did not significantly differ according to gender or length of aganglionosis. Overall, 43,4% of patients complained ophthalmologic issues (mostly refraction anomalies), 9,4% visual impairment, 20,7% congenital anomalies of the kidney and urinary tract, 4,7% congenital heart disease, 4,7% hearing impairment or deafness, 2,3% central nervous system anomalies, 8,5% chromosomal abnormalities or syndromes and 12,3% other associated anomalies.

**Conclusions:**

Our study confirmed the underestimation of certain associated anomalies in Hirschsprung patients, such as hearing impairment and congenital anomalies of the kidney and urinary tract. Subsequently, based on our results we strongly suggest performing renal US and audiometry in all patients. Conversely, ophthalmologic assessment and cerebral and heart US can be performed according to guidelines applied to the general population or in case of patients with suspected clinical features or chromosomal abnormalities. This updated diagnostic algorithm aims at improving overall outcome thanks to better prognostic expectations, prevention strategies and early rehabilitation modalities. The investigation of genetic background of patients with associated anomalies might be the next step to explore this intriguing multifactorial congenital disease.

## Background

Hirschsprung’s disease (HSCR) is a congenital multifactorial developmental disorder of the enteric nervous system characterized by the absence of ganglion cells in the hindgut with variable distal bowel involvement. This is a rare disease, which occurs with an incidence of 1 into 5000 live births as a consequence of premature arrest of cranio-caudal migration of neural crest derived neuroblasts (NCN) in the hindgut, and is therefore regarded as a neurocristopathy [1-3]. The neural crest is one of the earliest organs to form within the developing embryo, during formation of the neural tube. Cells of the neural crest are pluripotential and follow migratory pathways depending on their axial level of origin. NCN differentiate into crucial cell types ancestors and participate in the development of various organs such as adrenal medulla, melanocytes, craniofacial cartilage and bone, smooth muscle, neuroendocrine cells and sympathetic and parasympathetic nervous systems, including the enteric nervous system [3,4]. Alterations in any of the genes involved in the enteric nervous system development may interfere with the colonization process of NCN and represents a primary aetiology for HSCR [5,6]. The major gene responsible for HSCR is RET, whose mutations are detected in up to 50% of familial and in 7-35% of sporadic cases [7]. RET is crucial for the embryologic development of the enteric nervous system but also of other organs, including kidneys and urinary tract [8]. So far, a number of other minor HSCR susceptibility genes have been identified in less than 5% of patients [7]. The involvement of heterogeneous genetic pathways and pluripotential cell lineage explains why HSCR disease can be associated to malformations basically involving all organs and system.

Associated anomalies have been detected in between 20% and 30% of HSCR patients according to literature [5,9,10]. In 2006, Moore SW performed a systematic review of 18 series with 4328 patients reported in the last forty years and suggested an average percentage of associated anomalies of 21.1%, ranging between 5% and 35% [10]. Similarly, Amiel J and co-workers reported a percentage of associated anomalies of around 30% [9]. Nonetheless, both Authors suggested a measure of underestimation [9,10]. In accordance to these considerations, we recently demonstrated that Congenital Anomalies of the Kidney and Urinary Tract (CAKUT) occur 4-folds more frequently than previously reported [8]. As a consequence, it comes clear that the exact clinical phenotype of HSCR patients is yet unknown and deserves definition to provide our patients with reliable prognostic expectations. A better knowledge of all associated anomalies in HSCR will also reveal undisclosed genetic implications that will improve the comprehension of HSCR pathophysiology. We therefore implemented a prospective observational cross-sectional study on 106 consecutive HSCR patients aimed at defining the percentage of associated anomalies and at subsequently implementing an up-to-date diagnostic algorithm.

## Methods

All consecutive HSCR patients who were admitted to Giannina Gaslini Research Institute between January 2010 and December 2012 were eligible for this prospective observational cross-sectional study. Inclusion criterion was the presence of a reliable diagnosis of HSCR achieved with adequate pathological assessment [11]. Exclusion criteria were uncertain diagnosis and/or refusal to participate.

This study received Institution Review Board approval on the 15^th^ of October 2009. After enrolment, patients and parents were interviewed to collect demographic data, personal and family history and any information regarding known associated anomalies. All families were asked to sign a specific Informed Consent form and in case of acceptance each patient underwent an advanced non-invasive diagnostic algorithm, including ultrasound scan (US) of the kidney and urinary tract, cardiologic assessment with cardiac US, audiometry and ENT assessment, ophthalmologic assessment, cerebral US (< 1 year-old), and further specialist investigations based on clinical features. All these non-invasive investigations were performed according to widely accepted guidelines. The following data were collected according to the Personal Data Protection Act:

• Demographics

• Personal and family history.

• Length of aganglionosis [8].

** ○ S-HSCR** (aganglionosis extending up to the left descending colon).

** ○ L-HSCR** (aganglionosis extending beyond the splenic flexure, up to the ascending colon and/or caecum).

** ○ TCSA** (aganglionosis involving the whole colon).

** ○ TIA** (less than 20 cm of normoganglionic bowel).

• Associated anomalies detected during the phenotype screening.

• Other associated anomalies or syndromes not included in the phenotype screening.

### Details of phenotype screening

#### Cakut screening

All patients underwent renal US + urinalysis and colture. In case of positive findings we applied widely accepted diagnostic guidelines to properly investigate the patients [8]. To perform renal US we used Philips iU 22 US scanner (Philips, DA Best, The Netherlands) equipped with convex and linear multiple frequency electronic transducers, similarly to the study already published by our study group in 2009 [8]. We also used the same criteria to define all possible CAKUT variants: renal agenesis, renal dysplasia-hypoplasia, hydronephrosis, vesicoureteric reflux, duplex collecting system, and horseshoe kidney [8].

#### Cardiovacular screening

The cardiovascular screening included personal medical history, physical examination, non invasive blood pressure measurement, a twelve lead electrocardiogram and echocardiogram. Detailed US was performed with a pulsed, continuous, and color-Doppler provided US system (Philips iE33), using a 5 or 8 MHz transducer. Echo-scan was done by a transthoracic approach with the patient in the supine and left decubitus position. Morphological variables were measured offline by two observers (G.T. and M.D.) who used the so called Z score for normalization of the cardiac structures dimensions to the body size [12-14].

Cardiac anatomy was routinely assessed by a segmental approach that included the abdominal view, the subcostal short and long axis view, the apical four-chambers view, the parasternal short axis view at the level both of the papillary muscles and of the mitral valve, the parasternal long axis view and the suprasternal view. Two-dimensional echocardiographic measurements included the end-systolic diameter of the aortic annulus of the Valsalva sinuses and of the ascending aorta. These dimensions were measured from the parasternal long-axis view with the “inner edge convention”, which uses the innermost bright edge reflection as a contour. End-diastolic posterior wall thickness, left ventricular internal dimension and interventricular septal thickness were measured through the M-mode technique, which was performed also for calculating the shortening fraction. Diastolic ventricular function as well as valves function was assessed by both pulsed and continuous Doppler technique [15-18].

#### Auditory and ent screening

We assessed the possible presence of audiologic risk factors as those indicated by the Joint Committee of Infant Hearing, including in-utero infections, craniofacial anomalies, physical findings suggesting syndromes known to include a sensorineural or permanent conductive hearing loss, neonatal intensive care (NICU) admission longer than 5 consecutive days, exposure to ototoxic medications, family history and prematurity [19]. The tests used for auditory screening depended on patients’ age and degree of cooperation. Hearing impairment (HI) was defined in case of hearing threshold higher than 20 dB. Deafness or unilateral anacusia were defined in case of complete absence of response to auditory stimuli [20]. Screening was conducted in a quiet room without visual and auditory distractions according to widely accepted standards [21].

• From birth to 6 months of age - We used the Automated Auditory Brain System (AABR). This methodology measures cochlear response in the 1 to 4-kHz range with a broadband click stimulus in each ear. The automated screener provides a pass-fail report; no test interpretation by an audiologist is required. A “fail” report on an AABR implies a new non-automatic ABR test associated to ENT clinical assessment.

• From 6 months to 3 years of age - We used the Visual Reinforcement Audiometry (VRA), a behavioural test measuring responses of the child to speech and frequency-specific stimuli presented through speakers or insert earphones.

• From 3 years to 6 years of age - We used the Conditioned Play Audiometry (CPA) through earphones; in this test the child is conditioned to respond when stimulus tone is heard, such as to put a peg in a pegboard or drop a block in a box.

• From 6 years of age onwards - We used the Conventional Audiometry (CA) in which the patient is instructed to raise a hand in case of stimulus is heard. Deafness or hearing impairment were defined and graded according to widely accepted international standards.

#### Ophtalmologic screening

We recorded detailed information regarding family history and any risk factors for visual impairment, including length of stay in NICU and gestational age and weight at birth. Methodology differed according to the age. Visual impairment (VI) was defined as uncorrected visual acuity (VA) < 20/50 and was assessed only in patients older than 36 months of age. In younger patients we addressed strabismus or other VA indirect measures.

• Before 3 years of age, we performed only an objective evaluation due to the lack of child compliance. After a good visual inspection to exclude eye and eyelid malformations, anatomic abnormality, evident strabismus, anomalies of eye motility and head posture, we assessed:

 ○ Anterior segment and pupillary evaluations. The anterior segment was evaluated using a handheld or stand-mounted slit lamp. Pupillary responses were tested with a hand light.

 ○ Cycloplegic autorefraction and keratometry, with Plus Optix autorefractor (Plusoptix Inc., Atlanta, GA, USA) or Retinomax autorefractor, (Nikon, Melville, NY, USA). Refractory defects were defined and graded according to widely accepted international standards [22].

 ○ Fundus with cycloplegia.

• After 3 years of age, throughout adolescence and adulthood we also performed visual acuity assessment, orthoptist screening with distance acuity test, cover test, extrinsic ocular movements, prism test, and Lang-stereotest. Moreover, we also looked for the presence of amblyopia. In this age group we resorted to subjective components thanks to reasonably good patients’ compliance.

Patients were divided into three different age groups: younger than 3 (infants), between 3 and 6 (pre-school children), and older than 6 years of age (school children) to compare our results to literature data [22-24].

#### Brain screening and definition

Studies were performed with convex and linear-array 7,5 MHz transducers via the anterior fontanel in coronal and sagittal planes. Further screening images were obtained via other supplemental fontanels (posterior and mastoid). The investigation was performed in all patients younger than 12 months and/or with adequate echoic windows. We assessed subarachnoid spaces, lobes, ventricles and midline structures to rule out major congenital malformations [25].

#### Other associated anomalies and syndromes

Further investigations were performed basing on clinical features detected during the first detailed physical examination aimed at ruling out major gross abnormalities, including skull and facial malformations, cleft lip or palate, gross skeletal malformations, skin pigmentation disorders, external genitalia abnormalities, neurodevelopmental delay, etc. All information regarding other associated anomalies or syndromes reported by parents or patients were recorded. In case of need, patients were investigated in details according to widely accepted standards of care. In particular, chromosome analysis, array-CGH or direct sequencing of targeted genes was performed basing on genetist’s suggestion after appropriate dysmorphology assessment.

#### Statistical analysis

Descriptive statistics were reported as percentages with 95% confidence interval (CI), when indicated, in case of categorical variables. Median and range was used for age, given the wide variability in our series. Differences in the frequencies of each categorical variable were evaluated by the *Chi-square test.* Comparison of continuous data was performed using the 2*-tailed unpaired t test*. In case of scant data or non-normal distribution, non parametric tests (*Man-Whitney*) were used. A p value lower than 0.05 was considered as statistically significant. Analyses were performed using Stata for Windows statistical package (release 9.0, Stata Corporation, College Station, TX).

## Results

### Demographics

Overall, 144 consecutive HSCR patients were admitted to the Department of Surgery of Giannina Gaslini Institute during the study period. One-hundred-and-six (106) patients met the inclusion criteria, were enrolled and underwent the entire phenotype screening during the study period. Median age was 2,4 year (range 3 months to 25 years). Male to female ratio was 3,4:1. Nineteen patients (17,9%) had ultralong HSCR (either TCSA or TIA), 8 (7,6%) had L- HSCR, and 79 (74,5%) had S- HSCR. A total of 112 associated anomalies were detected in 57,5% (95% CI, 48,0-66,5%) of the patients (61 out of 106) with a proportion that did not significantly differ either according to length of aganglionosis or gender (*p* = *0,8209 and 1,0000*, respectively). Twenty-eight patients (26,4%) were already aware of their association when they were enrolled in the study (see Table 1 for details).

**Table 1:** Overall details of 106 HSCR who completed the screening during the study period

	**Overall, n (screened)**	**% (95% CI)**
**Patients, n**	106 (106)	
**Median age**	2,4	
**Male to female ratio**	3,4:1	
**VI or ophthalmological issues**	46 (106)	43,4% (95% CI, 34,4-52,9%)
**CAKUT**	22 (106)	20,7% (95% CI, 14,1-29,4%)
**CHD**	5 (106)	4,7% (95% CI, 2,0-10,6%)
**HI or deafness**	5 (106)	4,7% (95% CI, 2,0-10,6%)
**CNS abnormalities**	1 (43)	2,3% (95% CI, 0,4-12,1%)
**Other associated anomalies**	13 (106)	12,3% (95% CI, 7,3-19,9%)
**Syndromes**	9 (106)	9,4% (95%CI, 5,2-16,5%)
**Down Syndrome**	7 (106)	6,5% (95% CI, 3,2-13%)

**Legend**: *Syndrome,* Patients with known genetic abnormalities (i.e. Down Syndrome, Cat Eye, CCHS, etc.); *VI* = Visual Impairment; *CAKUT*, Congenital Anomalies of the Kidney and Urinary Tract; *CHD*, Congenital Heart Defect; *HI*, Hearing Impairment; *CNS*, Central Nervous System.

### Ophtalmologic abnormalities or visual impairment

None of the patients was diagnosed with blindness. No major ocular abnormalities were detected in our series of patients. No family history of severe visual impairment (VI) or blindness was recorded. Refractive error or ophthalmologic issues were detected in 43,4% (95% CI, 34,4-52,9%) of the patients (Table 2 for details). Male to female ratio was 3,6:1. Five patients had chromosomal abnormalities and 1 had Congenital Central Hypoventilation Syndrome (CCHS) known to be at higher risk for VI. We observed the following phenotypes: hyperopia (29,2% of the overall series), astigmatism (26,4%), myopia (7,5%), strabismus (4,7%), amblyopia (4,7%), papilla abnormalities (1,9%), opaque iris (0,9%) and severe ptosis (0,9%). Twenty-eight patients were diagnosed during the phenotype screening. Ophthalmologic abnormalities were observed in 34% of the patients younger than 3, in 48% of those aged between 3 and 6 and in 54% of those older than 6 years of age. Overall percentage of VI was 9,4%.

**Table 2:** Ophthalmologic abnormalities in 46 patients

**Pt**	**Sex**	**HSCR type**	**Age (m)**	**Ophthalmologic abnormality**	**Side**	**VI**	**Other anomalies**
1	F	TCSA	146	Mild myopia	R	N	
2	M	TCSA	12	Hyperopic astigmatism	B	N	
3	M	TCSA	32	Moderate myopia	B	N	FMTC; CAKUT
4	M	TCSA	107	Hyperopic astigmatism	B	N	GH DEFICIENCY
5	F	TCSA	16	Hyperopic astigmatism	B	N	GUT ATRESIA
6	F	L-HSCR	11	Hyperopic astigmatism	B	N	
7	F	L-HSCR	52	Severe hyperopic astigmatism	B	N	
8	M	L-HSCR	30	Mixed astigmatism + opaque iris	B	N	ONDINE; CAKUT; CRYPTO; HI
9	M	L-HSCR	62	Hyperopic anisometropia + amblyopia + ptosis	L	Y	DOWN
10	F	S-HSCR	5	Hyperopia	B	N	
11	M	S-HSCR	129	Hyperopic anisometropia + severe amblyopia	R	Y	CAKUT
12	F	S-HSCR	41	Hyperopia	B	N	
13	M	S-HSCR	3	Hyperopic anisometropia	L	N	
14	M	S-HSCR	37	Hyperopic astigmatism	R	N	
15	M	S-HSCR	70	Hyperopic astigmatism	B	N	
16	F	S-HSCR	26	Myopic astigmatic anisometropia	L	N	
17	F	S-HSCR	30	Hyperopic astigmatism	B	N	
18	M	S-HSCR	17	Hyperopic astigmatism	B	N	
19	M	S-HSCR	4	Hyperopia	L	N	
20	M	S-HSCR	133	Mixed astigmatism	B	N	FMTC
21	M	S-HSCR	5	Hyperopic astigmatism	B	N	
22	M	S-HSCR	69	Hyperopic astigmatism	L	N	
23	M	S-HSCR	76	Hyperopia	B	N	CAKUT
24	M	S-HSCR	26	Hyperopic astigmatism	B	N	CAKUT
25	M	S-HSCR	11	Hyperopic astigmatism	B	N	DOWN; CHD
26	M	S-HSCR	44	Hyperopic astigmatism + amblyopia	L	Y	
27	M	S-HSCR	76	Hyperopic astigmatism	B	N	CAKUT; HI
28	F	S-HSCR	248	Myopia	B	N	CAKUT; HI
29	M	S-HSCR	16	Diverging strabismus	B	Y	CAT EYE; CHD; CAKUT; VI; EAR PIT
30	M	S-HSCR	3	Hyperopic astigmatism	B	N	
31	M	S-HSCR	220	Mixed astigmatism	B	N	CAKUT
32	M	S-HSCR	54	Hyperopic astigmatism + mixed amblyopia	L	Y	
33	M	S-HSCR	161	Myopic astigmatism	L	N	CRYPTO; HYPERTG
34	M	S-HSCR	288	Vertical strabismus	L	Y	OSTEOPOROSIS
35	M	S-HSCR	113	Myopic astigmatism	B	N	
36	F	S-HSCR	290	Hyperopic astigmatism + strabismus	B	Y	DOWN; CHD; CAKUT
37	M	S-HSCR	77	Amblyopia	L	Y	
38	M	S-HSCR	152	Severe myopia + converging strabismus	L	Y	DOWN; CHD
39	M	S-HSCR	109	Hyperopic astigmatism	B	N	ADHD
40	M	S-HSCR	154	Myopia	B	N	CAKUT; DYSLESSIA; CCA
41	M	S-HSCR	4	Hyperopic astigmatism	B	N	
42	M	S-HSCR	101	Hyperopic astigmatism	B	N	
43	M	S-HSCR	49	Hyperopia	B	N	CAKUT
44	M	S-HSCR	138	Hyperopic anisometropia	B	N	
45	M	S-HSCR	142	Strabismus	B	Y	EARLY PUBERTY
46	M	S-HSCR	44	Hyperopic astigmatism	B	N	CAKUT

**LEGEND**: GH, Growth Hormone; **ADHD**, Attention Deficit Hyperactivity Disorder; **CCA**, Corpus Callosum Agenesis; **B**, Bilateral; **L**, Left; **R**, Right; **VI**, Visual impairment; **Y**, Yes; **N**, No.

Incidence of visual impairment was 9,4%.

### Cakut

CAKUT were detected in 20,7% (95% CI, 14,1-29,4%) of the patients. Male to female ratio was 4,5:1. One patient had a clear familial history of CAKUT. We observed a total of 26 abnormalities with a slight right-sided preponderance (right to left ratio = 1,14:1) (Table 3 for details). Three patients had two or more co-existing CAKUT. Thirteen patients were diagnosed during the phenotype screening. In 7 patients CAKUT were symptomatic with urinary tract infections requiring medications (antibiotics either for treatment or prophylaxis) and long-term follow-up. Three patients required surgery.

**Table 3:** Details of 22 patients with CAKUT

**ID**	**Sex**	**HSCR type**	**CAKUT**	**Side**	**Other anomalies**
1	M	TIA	MCDK	R	
2	F	TCSA	RH	B	HI
3	M	TCSA	RA	L	FMTC; VI
4	M	TCSA	VUR	B	
5	M	L-HSCR	RH	R	ONDINE; CRYPTO; VI; HI
6	M	S-HSCR	RH	R	VI
7	M	S-HCSR	DCS	L	VI
8	M	S-HCSR	HN	B	VI
9	M	S-HCSR	HN	L	PALATE CLEFT
10	M	S-HSCR	VUR	R	EAR PIT
11	F	S-HCSR	RH + DCS	B	VI; HI
12	M	S-HCSR	RH	L	VI; HI
13	M	S-HCSR	VUR	B	CAT EYE; CHD; VI; EAR PIT
14	M	S-HSCR	RH	R	VI
15	F	S-HSCR	RH	B	
16	M	S-HSCR	RH + HN	B	DOWN; CHD; VI
17	M	S-HSCR	VUR	R	
18	M	S-HSCR	HN	B	VI; DISLESSIA; CCA
19	M	S-HSCR	HN	L	VI
20	M	S-HSCR	VUR + PUV + RH	R	
21	F	S-HSCR	RH	R	VI
22	M	S-HSCR	VUR	L	DOWN; ATRESIA

**Legend:***TIA*, Total Intestinal Aganglionosis; *TCSA*, Total colonic aganglionosis; *L-HSCR*, Aganglionosis extended between left transverse colon and the cecum; *S-HSCR*, Aganglionosis extending up to the splenic flexure; *MCDK*, Multicystic Dysplastic Kidney; *RH*, Renal hypoplasia; *RA*, Renal Asymmetry; *HN*, Hydronephrosis; *VUR*, Vesicoureteric Reflux; *DCS*, Duplex Collecting System; *PUV*, Posterior urethral valve; *R*, Right; *B*, Bilateral; *L*, Left; *HI*, Hearing impairment; *FMTC*, Familiar Medullary Thyroid Carcinoma; *VI*, Visual Impairment; *CRYPTO*, Cryptorchidism; *CHD*, Congenital Heart Disease; *CCA*, Corpus Callosum Agenesis.

Sixteen right and 14 left rental units were involved for a slight right side preponderance. Most of HSCR patients with CAKUT (17/22 = 77%) had further associated malformations.

### Congenital heart diseases (CHD)

Major CHD were detected or confirmed during phenotype screening in 4,7% (95% CI, 2–10,6%) of the patients (Table 4 for details). Male to female ratio was 1,5:1. No positive family history was reported by any of the patients. CHD were represented by septal defects (either atrial or ventricular) in all patients but one. Four patients, all carrying chromosomal abnormalities, had severe CHD requiring surgical correction. In addition to the above-mentioned abnormalities, we detected *ostium secundum* type ASD in 4 patients and dilatation of the aortic sinus in 3. Those patients are being followed up in the long term. See Table 4 for details.

**Table 4:** Details of HSCR patients with CHD

**Pt ID**	**Sex**	**Age (m)**	**HSCR type**	**Disease**	**Management**	**Other anomalies**
1	F	12	TCSA	AC	Surgical intervention	TURNER
2	F	291	S-HSCR	ASD + VSD + MI	Surgical intervention	DOWN; CAKUT; VI
3	M	152	S-HSCR	ASD + VSD	Surgical intervention	DOWN; VI
4	M	15	S-HSCR	ASD + VSD	Surgical intervention	CAT EYE; CAKUT; VI; EAR PIT
5	M	3	S-HSCR	ASD + small VSD	F-UP	DOWN
	M	26	S-HSCR	AS DIL.	F-UP	CAKUT; VI
	M	52	L-HSCR	AS DIL.	F-UP	CAKUT; EAR PIT
	M	134	S-HSCR	AS DIL.	F-UP	
	M	95	TCSA	ASD (OS)	F-Up	
	M	161	S-HSCR	ASD (OS)	F-Up	VI; IPERTG; CRYPTO
	M	288	S-HSCR	ASD (OS)	F-Up	VI; OSTEOPOROSIS
	M	40	S-HSCR	ASD (OS)	F-Up	DOWN

**Legend:***AC* = Aortic Coarctation; *ASD* = Atrial Septal Defect; *VSD* = Ventricular Septal Defect; *MI* = Mitral Insufficiency; *F-UP* = Follow up; *AS DIL.* = Aortic Sinus Dilatation.

Five patients were diagnosed with major CHD (4.7%). Four patients required cardiac surgery. All patients with CHD suffered from chromosomal abnormalities (3 Down, 1 Cat Eye, and 1 Turner Syndrome). In addition to the above-mentioned abnormalities, we detected dilatation of the aortic sinus in 3 patients and ostium secundum type ASD in 4. All those patients are being followed up in the long term.

### Hearing impairment (HI), deafness or ent anomalies

HI or deafness were reported in 4,7% (95% CI, 2–10,6%) of the patients with slight female preponderance. Two further patients (1,9%) had congenital preauricular fistulas (Ear pit) (Table 5 for details). No positive family history was reported by any of the patients. We observed the following HI: 3 sensorineural hypoacusia, 1 mixed sensorineural/conductive hypoacusia and 1 unilateral deaf. No bilateral involvement was reported. One patient had CCHS known to be potentially associated to HI. Four patients were diagnosed during the phenotype screening. In addition to the above-mentioned anomalies we could detect bilateral deep at frequencies higher than 8000 Htz in 3 patients and conductive HI in 12, mostly related to upper airways infections or mucous retention.

**Table 5:** Patients with hearing impairment, deafness or ENT anomalies

**Pt**	**Sex**	**HSCR type**	**ORL disease**	**Side**	**Other anomalies**
1	F	TCSA	Sensorineural hypoacusia	L	CAKUT
2	M	L-HSCR	Sensorineural hypoacusia	B	ONDINE; CAKUT; VI; TESTIS
3	F	S-HSCR	Sensorineural hypoacusia	B	
4	M	S-HSCR	Preauricular fistula (Ear Pit)	L	CAT EYE; CAKUT; CHD; VI
5	M	S-HSCR	Anacusia	R	CAKUT; VI
6	M	S-HSCR	Preauricular fistula (Ear Pit)	L	CAKUT
7	F	S-HSCR	Mixed hypoacusia + conductive hearing loss	B	CAKUT; VI

Five patients (4.7%) had sensorineural hypoacusia or anacusia. Two further patients had minor ENT anomalies (Ear Pit). Six out of seven patients with auditory problems or ENT anomalies had other congenital abnormalities. Right to left ratio was 0,7:1 with slight left side preponderance.

### Central nervous system abnormalities

Corpus callosum agenesis was detected in 2,3% (95% CI, 0,4-12,1%) of the patients (1 out of 43 HSCR patients who underwent cerebral US). No other major brain abnormalities were detected. We identified ventricle asymmetry or subarachnoid spaces dilatation in 7 patients (16%) all with left side involvement. Moreover, we detected a mild hydromielic spinal cavity in a patient who underwent MRI for other reasons and congenital thalamic calcifications of unclear aetiology in another.

### Other associated anomalies

A further 18 associated anomalies have been detected in 12,3% (95% CI, 7,3-19,9%) of the patients from our series (12,3%). Amongst the various associated anomalies detected in our study outside the phenotype screening, endocrinologic and/or metabolic issues accounted for 4,7% of the patients (5/106), gastrointestinal abnormalities for 2,8% (3/106) and genital abnormalities and tumors (FMTC) for 1,9% each (2/106). One patient with severe developmental delay and one with corpus callosum agenesis underwent molecular genetic analysis of ZFHX1B/SIP1 to rule out Mowat-Wilson syndrome. See Table 6 for details.

**Table 6:** Other anomalies detected during the study

**ID**	**Sex**	**HSCR type**	**Associated anomalies**	**Screening results**
			**Chromosomal anomalies**	
1	F	TCSA	Down Syndrome	
2	F	TCSA	Turner Syndrome	CHD
3	M	L-HSCR	Down Syndrome	VI
4	M	S-HSCR	Down Syndrome	CHD; VI
5	F	S-HSCR	Down Syndrome	CHD; CAKUT; VI
6	M	S-HSCR	Down Syndrome	CHD; VI
7	M	S-HSCR	Down Syndrome	
8	M	S-HSCR	Down Syndrome	CAKUT
9	M	S-HSCR	Cat-Eye Syndrome	CHD; CAKUT; VI; HI
			**Metabolic issues**	
10	M	TCSA	GH deficiency	VI
11	M	S-HSCR	Familial Hyper-TG	VI
12	M	S-HSCR	Hypothyroidism	
13	M	S-HSCR	Osteoporosis	VI
14	M	S-HSCR	Precocious Puberty	VI
			**Gastrointestinal abnormalities**	
15	F	TCSA	Gut Atresia	VI
16*	M	TCSA	Coeliac Disease	
8	M	S-HSCR	Pancreas anularis	CHD; CAKUT
8	M	S-HSCR	Malrotation	CHD; CAKUT
			**Genital Abnormalities**	
17	M	L-HSCR	Cryptorchidism	CAKUT; HI; VI
11	M	S-HSCR	Cryptorchidism	VI
			**Tumors**	
18	M	TCSA	FMTC	CAKUT; VI
19	M	S-HSCR	FMTC	VI
			**Other Anomalies**	
16*	M	TCSA	Seizures	
17	M	L-HSCR	Ondine Syndrome	CAKUT; HI; VI
20	M	S-HSCR	Cleft Palate	CAKUT
21*	M	S-HSCR	Dyslessia	CAKUT; VI; CCA
22	M	S-HSCR	ADHD	VI

Most of these anomalies were already known and occurred in association with other associated anomalies in a syndromic fashion.

**Legend**: **FMTC** = Familial Medullary Thyroid Carcinoma; **ADHD**, Attention Deficit Hyperactivity Disorder; **CCHS**, Congenital Central Hypoventilation Syndrome; **CCA**, Corpus Callosum Agenesis; * = Mowat-Wilson excluded.

Four patients had more than one associated anomaly detected outside the screening (pts number 8, 11, 16 and 17). The great heterogeneity of organs and systems is evident in our series of patients. Only 4 of these patients had no further anomalies detected during the study.

### Syndromes

Chromosomal abnormalities or known syndromes (Down, Turner, Cat Eye, Congenital Central Hypoventilation Syndromes) have been detected in 9,4% (95% CI, 5,2-16,5%) of the patients (Table 6). The percentage of patients suffering from Down Syndrome in our series was 6,6% (95% CI, 3,2-13%). With regard to associated anomalies, syndromic HSCR patients proved to have a higher proportion of CHD compared to non-syndromic HSCR patients (p = 0,0001). Other associated anomalies have been detected more frequently in syndromic HSCR but the association proved not to be statistically significant (see Table 7 for details).

**Table 7:** Details of patients divided according to syndromic vs non-syndromic features

	**Syndromic, n (%, 95%CI)**	**Non-syndromic, n (%, 95% CI)**	***p***
**Patients, n**	10 (9%, 5%-16%)	96 (91%, 83%-95%)	
**Median age**	2,25	2,42	*n.a.*
**Male to female ratio**	2,33:1	3,57:1	*0,6912*
**S-HSCR**	6 (60%, 31%-83%)	73 (76%, 67%-83%)	*0,2719*
**Other HSCR forms**	4 (40%, 17%-69%)	23 (24%, 16%-33%)	
**VI**	6 (60%, 31%-83%)	40 (41,7)	*0,7918*
**CAKUT**	4 (40%, 17%-69%)	18 (18,7)	*0,2106*
**CHD**	**5 (50%, 24%-76%)**	**0 (0%, 0%-5%)**	***0,0001***
**HI**	1 (10%, 18%-40%)	4 (4%, 2%-10%)	*0,3968*
**CNS anomalies**	0 (0%, 0%-33%)	1 (1%, 0,2%-57%)	*1,000*
**OTHER**	3 (30%, 11%-60%)	10 (10%, 6%-18%)	*0,1044*

**Legend**: **n.a.**, Not assessed.

Although associated anomalies were encountered more frequently in syndromic patients, only CHD showed a statistically significant association.

### Multiple associations

Thirty-one patients (29,2%) had ≥ 2 associated anomalies or chromosomal abnormalities. Twelve (11,3%) had ≥ 3 and 3 (2,8%) had ≥ 4. The latter patients all had known syndromes (Down, Cat Eye, and Congenital Central Hypoventilation Syndrome).

## Discussion

This is the first prospective observational study aimed at defining the spectrum of possible phenotypes in a large cohort of HSCR patients. A reason why none previously addressed this issue probably relies on the rarity of the disease and on the difficulty to organize and perform such complex multidisciplinary prospective clinical assessments. In fact, literature reports regarding associated anomalies in HSCR are merely retrospective assessments of surgical series or systematic literature reviews [5,9,10].

The results of our study are remarkable and confirmed a measure of underestimation already suggested by some [5,8-10,26,27]. In particular, we demonstrated that the percentage of associated anomalies is higher than expected with involvement of basically all organs and systems.

Delayed diagnosis or mild symptoms can explain the reason for such a discrepancy. In fact, refractive anomalies or visual impairment (VI), mild CAKUT or unilateral hearing impairment (HI) can be diagnosed with significant delay in the absence of specific screening programmes [8,19,28].

With regard to phenotype considerations, our study confirmed the high percentage of CAKUT in HSCR patients, as previously published [8]. In fact, despite some overlap of patients included in both studies (17% of cases), the percentage of associated CAKUT remained constantly high as well as the preponderance of hypoplasia, vesicoureteric reflux and hydronephrosis amongst detected malformations. Based on the results of our studies, we thus confirm that renal US should be included as a routine diagnostic investigation in all HSCR patients.

We could not detect any major ocular anomaly in our series of HSCR patients but nearly 10% of VI and more than 40% of refraction abnormalities. The latter are frequent in HSCR patients as well as in the general population. In 2009, the Baltimore Pediatric Eye Disease Study indicated in 0,7% the prevalence of myopia and nearly 9% that of hyperopia, in 1030 white Americans younger than 6 years of age [22]. Similarly, in 2011 the Sydney Pediatric Eye Disease Study examined 2461 children younger than 6 years of age and suggested an incidence of 6,4% of VI mostly due to refractive errors, amblyopia, and strabismus [23]. In 2008 Jobke and co-workers performed a population-based study on 516 German children, adolescent and young adults aged between 2 and 35 years and ended up with a prevalence of over 75% of emmetropia, nearly 20% myopia and 5% hyperopia [24]. Due to the heterogeneity of literature data regarding the general population, we can hardly determine the relative risk of HSCR patients of having refraction abnormalities. Nonetheless, based on embryologic considerations regarding the shared neural crest origin of enteric nervous system and specific ocular structures and on the relatively high percentage of patients with refractive errors and VI observed in our study, we strongly recommend to perform routine ophthalmologic assessments (as those performed in the general population) in order to early detect and prevent VI, in accordance to WHO suggestions [28,29].

The prevalence of major or moderate CHD has been reported to be around 7,2 per 1000 live birth in the general population [30], whereas the proportion of CHD in HSCR patients has been reported to be around 5%, according to literature [5,9,10]. We could basically confirm these data (4,7% in our series). In particular, CHD detected during phenotype screening mostly occurred in patients with chromosomal abnormalities and were essentially represented by septal defects. This is in accordance with the embryologic role of NCN in the development of both enteric nervous system and cardiac outflow septation [31]. On the other hand, none of the patients presented conotruncal heart defects, whose pathogenesis is similarly related to the abnormal neural crest cell proliferation and migration [31]. The percentage of minor CHD or trivial lesions without clinical significance in our series accounted for another 6,6% of patients, well within literature ranges [30]. Based on these results we do suggest performing cardiac US only in the subpopulation of known or suspected syndromic HSCR patients.

In the general population, the incidence of HI in subjects without audiological risk factor is around 1,5‰, whereas that of subjects with audiological risk factors is 4,5% [32,33]. With specific regard to HSCR patients, syndromic HI can be found in patients with Waardenburg-Shah type 4, due to mutations of SOX10 gene [5,9,10]. Of note in 1973 Skinner described 4 patients with congenital deafness and HSCR and suggested that this association could not be fortuitous but based on shared embryological background as only one case had known risk factors for HI (streptomycin administration) [34]. Based on these considerations and on the results of our study (4,7% of HI), we recommend that HSCR itself should be included amongst risk factors for HI. Subsequently HSCR patients should undergo specific audiologic screening programmes as those suggested by the Joint Committee of Infant Hearing in 2007 [19].

Although Moore reported a relatively high proportion of brain abnormalities in HSCR patients, we could only confirm a case of corpus callosum agenesis in a patient of ours who was already diagnosed with prenatal US [10,26]. The child had no further syndromic features and a near-normal neurologic development (mild dyslexia). In our series of patients we could also detect some ventricle asymmetry and subarachnoid spaces dilatations that were found in less than 20% of cases, mostly on the left hand side. Of note, asymmetry between the sizes of the ventricles can be observed in up to 40% of healthy infants, being the left ventricle often larger than the right [25]. Based on these results and in accordance with literature data, we do not recommend performing routine cerebral US in all HSCR patients but only in those with clear syndromic features who have higher likelihood of carrying cerebral malformations (i.e. Mowat-Wilson Syndrome) [10,26,35].

Amongst other associated anomalies (Table 6) tumors were exclusively represented by FMTC, well known to be associated with specific genotypes at the RET gene locus in HSCR patients [7]. As expected, after testing for mutations of the RET gene (data not shown), we could verify that the two patients of our panel who are affected with HSCR + FMTC syndromic association are indeed carriers of the p.C609Y and p.Y791F mutations, respectively. These mutations have already been reported in association with this combined phenotype and also described as “Janus mutations” [36].

The percentage of anomalies that could have been tracked down retrospectively by notes reviews or questionnaire administration resembles that of literature reports [5,9,10]. In fact, 26,4% of patients was already aware of their association when they entered our screening programme. This aspect further confirms the reliability of our results and that a deeper clinical screening could have detected the missed associated anomalies (“the more you look for, the more you find”).

Besides providing a novel view on clinical associations and variable symptoms of HSCR disease in children, the present data prompt us to deepen into follow-up data from adults who were treated for HSCR, which are lacking at the moment. Indeed, we cannot exclude that additional organs or systems can be involved and become impaired later in adult life. Indeed, typical adulthood associated diseases have not been described yet and are unknown at the moment. For instance, association with specific cancers, nervous degeneration, intestinal chronic inflammation and/or other late-onset disorders cannot be ruled out and further investigation in an adult set of HSCR patients would be advisable.

Our Institution is known to be a referral centre for HSCR with an intrinsic subsequent risk of inclusion BIAS. This concern was confirmed by the higher than expected percentage of ultralong forms of the disease in our series (17,9%). Nonetheless, given the fact that the percentage and type of associated anomalies did not correlate to gender or length of aganglionosis, the results of our study maintained their strength. Furthermore, the percentage of Down Syndrome as well as the strong male preponderance observed in our series of patients are coherent with literature data and confirm that the results of our study are representative of the whole HSCR population [5,9,10].

The relatively small population of this observational study represents another potential limitation. As a consequence, the estimates we could provide have the intrinsic limitation of a wide variability and should be taken with care (see Tables 1 and 7 for details). Nonetheless, HSCR is a rare disease and the number of patients enrolled in such a relatively small time-span implies strong commitment and a multidisciplinary approach that deserve consideration.

**Figure 1:**
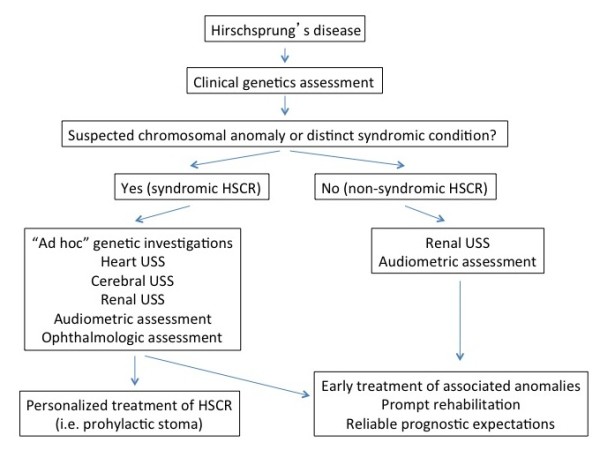
**Proposed diagnostic workup for patients with a reliable diagnosis of Hirschsprung’s disease.** Algorithm changes according to the present of sure or suspected chromosomal abnormalities.

It is evident that the implementation of a prospective multicentre research project is warranted. In fact, a larger series of patients could increase the strength of the results and possibly confirm the cost-effectiveness of this proposed diagnostic algorithm for a significant change in clinical practice for HSCR management (Figure 1).

## Conclusions

Based on the results of our study, we suggest performing US of the kidney and urinary tract as well as audiologic investigation in all cases, whereas heart US, cardiologic assessment, cerebral US and ophthalmologic assessment should be performed basing on clinical features and according to the standards of care adopted for the general population. On the other hand, all investigations should be considered for patients with known or suspected syndromes or chromosomal abnormalities in order to promptly apply specific prevention strategies, as we already suggested (i.e. preoperative prophylactic stoma and postoperative rectal irrigations in case of associated CHD) [37]. Figure 1 shows a suggested diagnostic algorithm. It includes early clinical genetic assessment in order to detect symptoms and signs suggestive of syndromes that would lead to further investigations.

Early diagnosis of associated anomalies provides more reliable prognostic expectations, prompt establishment of prevention strategies and implementation of adequate rehabilitation treatments. In accordance to our previous publication and to present results, we hypothesize that intestinal aganglionosis in HSCR patients represents the intestinal phenotype of a more complex syndrome driven by the interaction of neural crest maldevelopment and predisposing genetic background [5,7-10,29,31]. The investigation of genetic background of individuals presenting with associated anomalies might be the next step to explore this intriguing multifactorial congenital disease.

## Abbreviations

HSCR: Hirschsprung; NCN: neural crest derived neuroblasts; CAKUT: Congenital anomalies of the Kidney and Urinary Tract; US: Ultrasound scan; ENT: Ear nose and throat; NICU: Neonatal intensive care unit; HI: Hearing impairment; AABR: Automated auditory brainstem response; VRA: Visual reinforcement audiometry; CPA: Conditioned play audiometry; CA: Conventional audiometry; VI: Visual impairment; VA: Visual acuity; CCHS: Congenital central hypoventilation syndrome; CHD: Congenital heart disease.

## Competing interests

The authors declare that they have no competing interests.

## Authors’ contributions

APP conceived the study, supervised clinical screening and drafted the manuscript. IC, DM, PG, VJ, PB, GM participated in the acquisition of most of clinical data and in drafting the manuscript. VR, MM, CH participated in the acquisition of clinical data. GT, MD, MM, GM, CG, GG, EP, LS, AP participated in the design of the study and in the acquisition of clinical data. FL performed the statistical analysis and participated in drafting the manuscript. All authors read and approved the final manuscript.
